# Triplet versus doublet neoadjuvant chemotherapy regimens for locally advanced gastric cancer: a propensity score matching analysis

**DOI:** 10.1186/s12885-021-09093-9

**Published:** 2021-12-13

**Authors:** Yonghe Chen, Jiasheng He, Dan Liu, Jian Xiao, Xijie Chen, Haijie Tang, Dandong Luo, Chenyu Shang, Lei Lian, Junsheng Peng

**Affiliations:** 1 Department of Gastric Surgery, The Sixth Affiliated Hospital, Sun Yat-sen University, 26 Yuancun Erheng Road, Guangzhou, 510655 China; 2 Guangdong Institute of Gastroenterology, Guangdong Provincial Key Laboratory of Colorectal and Pelvic Floor Diseases, Guangzhou, 510655 China; 3 Department of Laboratory Science, The Second Affiliated Hospital, Guangzhou University of Chinese Medicine, Guangzhou, 510105 China; 4 Department of Medical Oncology, The Sixth Affiliated Hospital, Sun Yat-sen University, Guangzhou, 510655 China

**Keywords:** Gastric cancer, Neoadjuvant chemotherapy, Survival, Propensity score matching

## Abstract

**Background:**

To investigate the differences between doublet and triplet neoadjuvant chemotherapy (NAC) regimens in efficacy and safety profile.

**Methods:**

A total of 227 locally advanced gastric cancer (LAGC) patients who received NAC and sequential radical gastrectomy were reviewed. After propensity score matching (PSM), 140 patients with similar baseline characteristics were selected. Among them, 70 received doublet NAC regimens consisted of platinum and fluorouracil; the other 70 received triplet NAC regimens consisted of docetaxel, platinum, and fluorouracil.

**Results:**

The efficacy of doublet and triplet regimens was comparable after propensity score matching in terms of tumor regression (pathological complete response, Doublet 11.4% vs. Triplet 15.7%, *p* = 0.642), achieving of R0 resection (Doublet 88.6% vs. Triplet 88.6%, *p* = 1), 1-year disease-free survival (DFS) (Doublet 77.1% vs. Triplet 68.6%, *p* = 0.178), 3-years overall survival (OS) (Doublet 54.3% vs. Triplet 60.9%, *p* = 0.941). Post-surgery complications were more common in the triplet cohort (Doublet 5.7% vs. Triplet 27.1%, *p* = 0.001), especially abdominal infection (Doublet 0% vs. Triplet 11.1%, *p* = 0.001).

**Conclusions:**

A more intense preoperative triplet NAC regimen does not bring extra downstage effect and survival benefit compared to a doublet regimen. It may even result in a higher risk of post-surgery complications.

## Background

Gastric cancer is the fifth most common malignancy globally and the third leading cause of cancer-related death [1]. Most patients are diagnosed at the advanced stages of the disease, with an extremely poor prognosis [2, 3]. For operable locally advanced gastric cancer (LAGC), neoadjuvant chemotherapy (NAC) is recommended. However, the recommended regimens vary vastly in different guidelines [4–7]. Despite the variety of recommendations, all recommended regimens fall into two categories: the doublet regimens and the triplet regimens. Generally, the Asian guidelines endorse doublet regimens that combine platinum with fluorouracil or oral fluorouracil derivant (capecitabine or S-1). The other guidelines, such as the European Society for Medical Oncology (ESMO) and the National Comprehensive Cancer Network guideline (NCCN), recommend triplet regimens that combine docetaxel, platinum, and fluorouracil, such as the FLOT and the DCF regimen. In recent years, the FLOT regimen has become worldwide popular after the FLOT4 trial demonstrated its superiority over the classic ECF regimen [8]. Researchers proposed that adding docetaxel would significantly improve tumor downstaging, R0 resection rate, and survival [9, 10]. While some other researchers questioned that the addition of docetaxel only increases the toxicity, and its superiority over the modernized doublet regimen such as SOX or CAPOX, is yet to be proven [11]. Till now, a direct comparison between doublet and triplet NAC regimens for LAGC is lacking.

Hence, in this study, we aimed to determine the efficacy and toxicity of doublet and triplet regimens by adding real-world evidence of neoadjuvant chemotherapy for LAGC. We reviewed 227 cases of LAGC patients who received NAC and sequential resection surgery. Their tumor regression grade (TRG), R0 resection rate, toxicity, post-surgery complications and survival were retrospectively compared. We believe the results yielded from this study could provide valuable information for oncologists in choosing treatment strategies for LAGC patients.

## Methods

### Study design, inclusion, and exclusion criteria

This retrospective cohort study aimed to compare the efficacy and safety of doublet and triplet NAC regimens for LAGC patients. All the clinical data were retrieved from the Gastric Cancer Database of The Sixth Affiliated Hospital, Sun Yat-sen University (Guangzhou, China). All patients were followed up via re-examinations in the outpatient clinic and by telephone until mortality due to any reasons or loss of follow-up.

The inclusion criteria were as follows: (i) age between 18 and 80 years old with any gender; (ii) histological diagnosis of gastric/esophagogastric junction adenocarcinoma; (iii) received NAC and sequential radical gastrectomy; (iv) a clinical stage of T2-4N1-3M0; (v) Eastern Cooperative Oncology Group (ECOG) score 0–1.

The exclusion criteria were as follows: (i) received concurrent neoadjuvant radiotherapy or target therapy; (ii) less than 12 months of follow-up; (iii) insufficient staging information or uncertainty of distance metastasis; (iv) secondary malignant tumor.

### Pre-intervention staging, neoadjuvant chemotherapy regimen, and surgery

Before starting the treatments, all patients received enhanced thoracic-abdominal-pelvic computed tomography (CT) scan and/or endoscopic ultrasonography. All the clinical data were reviewed by the gastric cancer multi-disciplinary team consisting of surgeons, oncologists and radiologists. The recommendations of NAC were based on guidelines and the patients’ will, the NAC regimen was selected according to the recommendation of guidelines and the physicians’ preference. In this study, all the CT image sets were retrieved and re-assessed according to the 8th AJCC staging manual [12].

Triplet NAC regimens used in this study included FLOT and DCF. Doublet regimens used in this study included SOX, CAPOX, and FOLFOX. The details and dose intensity of the regimens are depicted below:

1. FLOT: docetaxel 50 ~ 60 mg/m^2^, oxaliplatin 85 mg/m^2^, and fluorouracil 2800 mg/m^2^; every 2 weeks;
2. DCF: docetaxel 50 mg/m^2^, cisplatin 50 mg/m^2^, and fluorouracil 2800 mg/m^2^; every 2 weeks;
3. SOX: oxaliplatin 130 mg/m^2^, tegafur gimeracil oteracil potassium capsule 40–60 mg bid Day1-Day14; every 3 weeks;
4. CAPOX: oxaliplatin 130 mg/m^2^, capecitabine 1000 mg/m^2^ bid Day1-Day14; every 3 weeks;
5. FOLFOX: oxaliplatin 85 mg/m^2^, fluorouracil 2800 mg/m^2^; every 2 weeks.

After the completion of NAC, the resectability of the primary tumor was confirmed by the multi-disciplinary team. All patients enrolled received curative tumor resection (total or subtotal gastrectomy) with standard D2 lymphadenectomy. A throughout examination of the abdominal cavity was routinely performed to determine the status of peritoneum metastasis before the resection.

### Pathological response, toxicity, and post-surgery complications evaluation

All resected specimens were examined to determine pathological stages and histological response to NAC. Tumor regression grades (TRG) were determined by the number of viable tumor cells that remained in the tumor, according to the Ryan standard [13, 14]. Grade 0 (complete response): no tumor cells remained; Grade 1 (major response): scattered single tumor cells remained; Grade 2 (moderate response): clustered tumor cells remained with fibrosis; Grade 3 (minor response): extensive tumor cells remained. Neoadjuvant chemotherapy related toxicity was evaluated according to the Common Terminology Criteria for Adverse Events version 5.0 [15]. Post-surgery complications were graded according to the Clavien-Dindo classification system [16]. Grade 2–4 complications, which mean complications that required medical or surgical interventions, were recorded.

### Follow-up

Following completion of the treatment, follow-up studies were conducted once every two months in the first six months and then once every three months, subsequently. Each follow-up study included medical history, physical examination, routine blood tests, comprehensive biochemical examinations, thoracic-abdominal-pelvic CT scan, and superficial lymph node B-ultrasonography.

### Propensity score matching

A propensity score matching (PSM) method was used for the patients enrolled in this study to select matching pairs with similar baseline characteristics in the two cohorts [17]. The matching factors were sex, age, tumor location, differentiation, diabetes, clinical T/N stage, and clinical stage groupings. The matching ratio was 1:1, and the caliper was 0.01. The matched pairs were divided into the doublet cohort and the triplet cohort.

### Data analysis

The normality of data was assessed using the Kolmogorov-Smirnov test and normal probability plots. Parameters that were not normally distributed were expressed in the median (upper quartile to lower quartile) and were analyzed using non-parametric tests: Mann-Whitney test or Kruskal–Wallis test, as appropriate. Normally distributed parameters were expressed in the form of mean ± standard deviation and were analyzed by Student’s *t*-test. Categorical variables were analyzed by the chi-square test. The survival difference was compared using the Kaplan-Meier method, and the hazard ratios were calculated in the Cox regression model. A *p*-value< 0.05 was identified as statistically significant. All statistical analyses were performed using the SPSS software version 25.0 (IBM, Armonk, NY, USA) and the R software version 4.0.2 (The R Foundation for Statistical Computing, Vienna, Austria; www.r-project.org).

## Results

### Patients characteristics

**Fig. 1:**
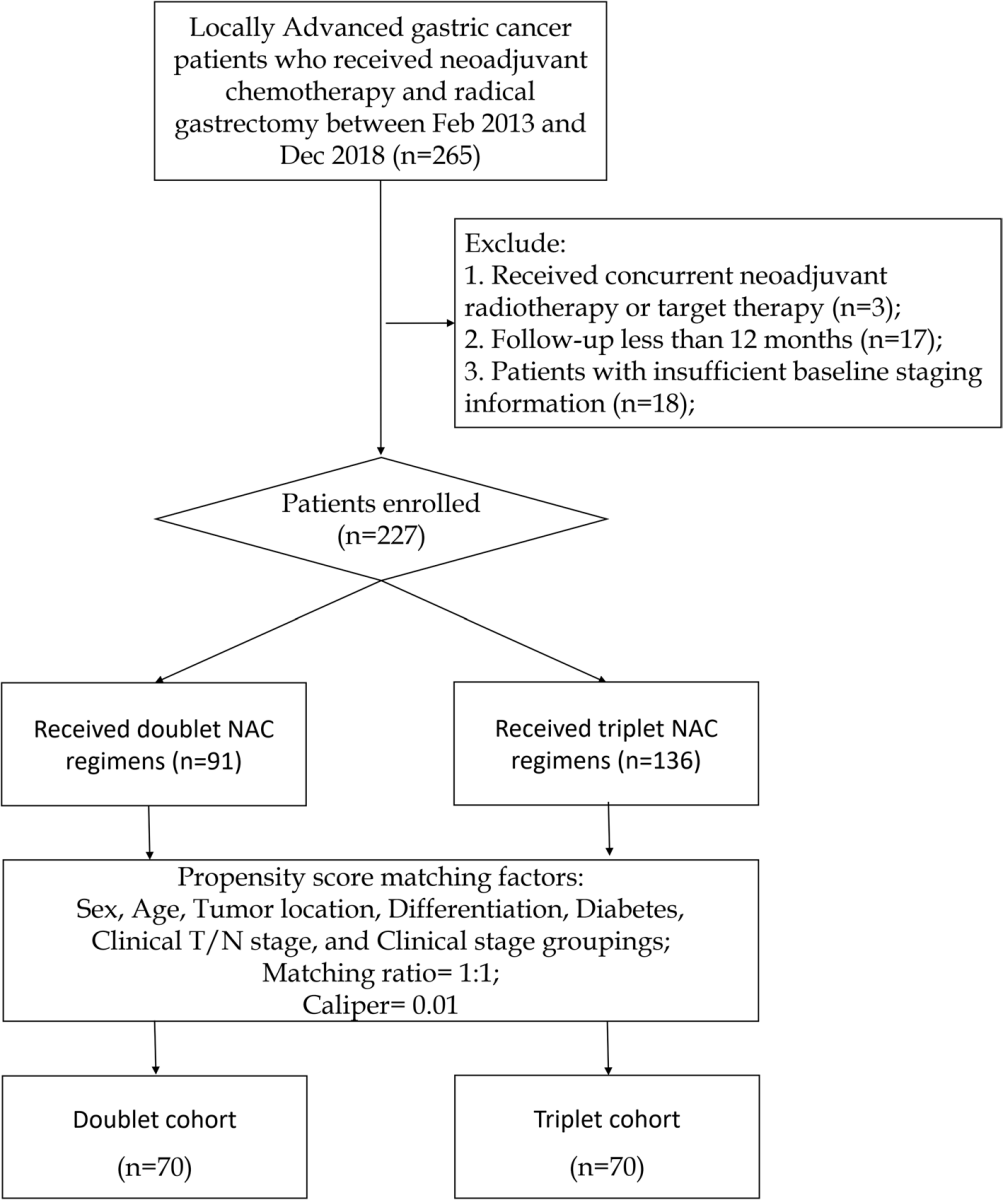
The flowchart showing the process of patients’ enrollment and propensity score matching

From February 2013 to December 2018, 265 eligible patients who received neoadjuvant chemotherapy and D2 radical gastrectomy were identified. As depicted in Fig. 1, 227 patients were included in the study after the screening, of which 91 received doublet regimen and 136 received triplet regimen for NAC. After propensity score matching, 140 patients with locally advanced lesions and similar characteristics were selected, with 70 patients in each cohort.

As shown in Table 1, baseline characteristics in the two study cohorts were similar. Patients were mostly male, with a median age of 60 years, and the tumor mainly was poorly differentiated adenocarcinoma. However, before PSM, patients in the triplet cohort were in significantly more advanced clinical stages, especially the cN stage. After PSM, both Clinical T and N stages were almost identical in both cohorts.

**Table 1:** Patients’ characteristics before and after propensity score matching (PSM)

Characteristic	Before PSM				After PSM			
	All *n* = 227 (%)	Double-agent *n* = 91 (%)	Triple-agent *n* = 136 (%)	*p*-value	All *n* = 140 (%)	Double-agent *n* = 70 (%)	Triple-agent *n* = 70 (%)	*p*-value
**Sex**								
*Male*	173 (76.2)	68 (74.7)	105 (77.2)	0.786	107 (76.4)	53 (75.7)	54 (77.1)	1
*Female*	54 (23.8)	23 (25.3)	31 (22.8)		33 (23.6)	17 (24.3)	16 (22.9)	
**Age**	59 [50,64]	60 [50,64]	59 [49,64]	0.398	59[49, 64.25]	59.5 [52.25, 64]	56 [48.25, 66]	0.347
*≤ 60 years*	124 (93.8)	48 (92.3)	76 (94.9)	0.684	78 (55.7)	38 (54.3)	40 (57.1)	0.865
*> 60 years*	103 (6.2)	43 (7.7)	60 (5.1)		62 (44.3)	32 (45.7)	30 (42.9)	
**Diabetes mellitus**								
*Yes*	16 (7)	5 (5.5)	11 (8.1)	0.485	12 (7.9)	5 (6.7)	7 (9.1)	0.57
*No*	211 (93)	86 (94.5)	125 (91.9)		140 (92.1)	70 (93.3)	70 (90.9)	
**Location**								
*Upper*	87 (38.3)	28 (30.8)	59 (43.4)	0.121	50 (35.7)	25 (35.7)	25 (35.7)	1
*Middle*	49 (21.6)	20 (22.0)	29 (21.3)		34 (24.3)	17 (24.3)	17 (24.3)	
*Lower*	91 (40.1)	43 (47.3)	48 (35.3)		56 (40.0)	28 (40.0)	28 (40.0)	
**Differentiation of adenocarcinoma**								
*Well*	7 (3.1)	3 (3.3)	4 (2.9)	0.981	–	–	–	1
*Moderately*	66 (29.1)	26 (28.6)	40 (29.4)		39 (27.9)	20 (28.6)	19 (27.1)	
*Poorly*	154 (67.8)	62 (68.1)	92 (67.6)		101 (72.1)	50 (71.4)	51 (72.9)	
**Clinical T stage**								
*T2*	4 (1.8)	2 (2.2)	2 (1.5)	0.512	2 (1.4)	1 (1.4)	1 (1.4)	0.998
*T3*	141 (62.1)	61 (67.0)	80 (58.8)		87 (62.1)	43 (61.4)	44 (62.9)	
*T4a*	55 (24.2)	20 (22.0)	35 (25.7)		35 (25.0)	18 (25.7)	17 (24.3)	
*T4b*	27 (11.9)	8 (8.8)	19 (14.0)		16 (11.4)	8 (11.4)	8 (11.4)	
**Clinical N stage**								
*N0*	2 (0.9)	2 (2.2)	0 (0.0)	< 0.001	–	–	–	0.983
*N1*	81 (35.7)	48 (52.7)	33 (24.3)		61 (43.6)	31 (44.3)	30 (42.9)	
*N2*	97 (42.7)	29 (31.9)	68 (50.0)		55 (39.3)	27 (38.6)	28 (40.0)	
*N3*	47 (20.7)	12 (13.2)	35 (25.7)		24 (17.1)	12 (17.1)	12 (17.1)	
**Clinical stage groupings**								
*IIA*	4 (1.8)	2 (2.2)	2 (1.5)	0.22	2 (1.4)	1 (1.4)	1 (1.4)	1
*IIB*	2 (0.9)	2 (2.2)	0 (0.0)		–	–	–	
*III*	194 (85.5)	79 (86.8)	115 (84.6)		122 (87.1)	61 (87.1)	61 (87.1)	
*IVA*	27 (11.9)	8 (8.8)	19 (14.0)		16 (11.4)	8 (11.4)	8 (11.4)	
**Regimen**								
*FOLFOX6*	8 (3.5)	8 (8.8)	–		4 (3.5)	4 (5.7)	–	
*CAPOX*	12 (5.3)	12 (13.2)	–		7 (5.3)	7 (10.0)	–	
*SOX*	71 (31.3)	71 (78.0)	–	–	59 (31.3)	59 (84.3)	–	–
*DCF*	5 (2.2)	–	5 (3.7)		4 (2.2)	–	4 (5.7)	
*FLOT*	131 (57.7)	–	131 (96.3)		66 (57.7)	–	66 (94.3)	
**Cycles**	4.00[4.00,4.00]	4.00[3.00,4.00]	4.00[4.00,5.00]	< 0.001	4.00[4.00,4.00]	4.00[3.00,4.00]	4.00[4.00,5.00]	0.006

### Neoadjuvant chemotherapy and toxicity

The FLOT and SOX were the mainstream regimens in our study, which took up 90% of the total sample. Other regimens included the FOLFOX, CAPOX, and the DCF regimen, all are commonly used regimens in clinical practice. All patients received a median of 4 cycles of NAC before surgery. The toxicity profiles were depicted in Table 2. Before and after PSM, the triplet cohort had a higher incidence rate of neutropenia and anemia, while thrombocytopenia was more common in the doublet cohort. The incidence rate of grade 3/4 hematological toxicity was not significantly different.

**Table 2:** Hematological toxicity according to the CTCAE 5.0

Characteristic	Before PSM				After PSM			
	All *n* = 227 (%)	Double-agent *n* = 91 (%)	Triple-agent *n* = 136 (%)	*p*-value	All *n* = 140 (%)	Double-agent *n* = 70 (%)	Triple-agent *n* = 70 (%)	*p*-value
**Overall grade 3/4 hematological toxicity**	130 (57.3)	47 (51.6)	83 (61.0)	0.206	75 (53.6)	33 (47.1)	42 (60.0)	0.175
**Anemia**								
*Grade 1*	35 (15.4)	22 (24.2)	13 (9.6)	0.016	18 (12.9)	14 (20.0)	4 (5.7)	0.044
*Grade 2*	81 (35.7)	28 (30.8)	53 (39.0)		53 (37.9)	23 (32.9)	30 (42.9)	
*Grade 3*	69 (30.4)	23 (25.3)	46 (33.8)		45 (32.1)	20 (28.6)	25 (35.7)	
*Grade 4*	24 (10.6)	8 (8.8)	16 (11.8)		11 (7.9)	4 (5.7)	7 (10.0)	
**Neutropenia**								
*Grade 1*	30 (13.2)	12 (13.2)	18 (13.2)	0.024	20 (14.3)	9 (12.9)	11 (15.7)	0.497
*Grade 2*	68 (30.0)	31 (34.1)	37 (27.2)		50 (35.7)	27 (38.6)	23 (32.9)	
*Grade 3*	50 (22.0)	26 (28.6)	24 (17.6)		30 (21.4)	16 (22.9)	14 (20.0)	
*Grade 4*	30 (13.2)	5 (5.5)	25 (18.4)		14 (10.0)	4 (5.7)	10 (14.3)	
**FebrileNeutropenia**								
*Grade 0*	220 (96.9)	90 (98.9)	130 (95.6)	0.306	134 (95.7)	69 (98.6)	65 (92.9)	0.211
*Grade 3*	7 (3.1)	1 (1.1)	6 (4.4)		6 (4.3)	1 (1.4)	5 (7.1)	
**Thrombocytopenia**								
*Grade 1*	38 (16.7)	18 (19.8)	20 (14.7)	< 0.001	24 (17.1)	15 (21.4)	9 (12.9)	< 0.001
*Grade 2*	33 (14.5)	27 (29.7)	6 (4.4)		25 (17.9)	21 (30.0)	4 (5.7)	
*Grade 3*	18 (7.9)	10 (11.0)	8 (5.9)		9 (6.4)	6 (8.6)	3 (4.3)	
*Grade 4*	4 (1.8)	0 (0.0)	4 (2.9)		2 (1.4)	0 (0.0)	2 (2.9)	
**Creatinine elevation**								
*Grade 1*	24 (10.6)	12 (13.2)	12 (8.8)	0.606	17 (12.1)	10 (14.3)	7 (10.0)	0.260
*Grade 2*	7 (3.1)	2 (2.2)	5 (3.7)		6 (4.3)	1 (1.4)	5 (7.1)	
*Grade 3*	1 (0.4)	0 (0.0)	1 (0.7)		1 (0.7)	0 (0.0)	1 (1.4)	
*Grade 4*	1 (0.4)	0 (0.0)	1 (0.7)		1 (0.7)	0 (0.0)	1 (1.4)	
**Alanine transaminase elevation**								
*Grade 1*	130 (57.3)	62 (68.1)	68 (50.0)	0.007	87 (62.1)	48 (68.6)	39 (55.7)	0.253
*Grade 2*	20 (8.8)	7 (7.7)	13 (9.6)		12 (8.6)	5 (7.1)	7 (10.0)	
*Grade 3*	19 (8.4)	5 (5.5)	14 (10.3)		10 (7.1)	4 (5.7)	6 (8.6)	
*Grade 4*	3 (1.3)	3 (3.3)	0 (0.0)		2 (1.4)	2 (2.9)	0 (0.0)	

Abbreviations: *PSM* Propensity score matching; *CTCAE* Common Terminology Criteria for Adverse Events

### Surgery, pathological findings, and complications

As depicted in Table 3, no perceptible differences were observed in terms of the R0 resection rate between the two groups (Doublet 90.1%, 82/91 vs. Triplet 88.2%, 120/136, *p* = 0.829). After PSM analysis, the R0 resection rate was similar in the doublet group and the triplet cohort (both 88.6%, 62/70, *p* = 0.1). The incidence rate of complications (Clavien-Dindo grade 2–4) was significantly higher in the triplet cohort (before PSM: Doublet 8.8%, 8/91 vs. Triplet 27.2%, 37/136, *p* = 0.001; after PSM: Doublet 5.7%, 4/70 vs. Triplet 27.1%, 19/70, p = 0.001), especially surgery-related abdominal infections, caused mainly by anastomotic leakage. As for pathological findings, TRG in the two cohorts was statistically similar whether it was before or after the PSM analysis. The pathological complete response (grade 0: before PSM, Doublet 11.0%, 10/91 vs. Triplet 16.2%, 22/136, *p* = 0.686; after PSM, Doublet 11.4%, 8/70 vs. Triplet 15.7%, 11/70, *p* = 0.642) was higher in the triplet cohort, but the difference was insignificant. Others were similar, including the post-surgical ypTN stages spectrum and numbers of positive/total harvested lymph nodes.

**Table 3:** Surgical outcomes, pathological findings, and adjuvant chemotherapy

Characteristic	Before PSM				After PSM			
	All *n* = 227 (%)	Double-agent *n* = 91 (%)	Triple-agent *n* = 136 (%)	*p*-value	All *n* = 140 (%)	Double-agent *n* = 70 (%)	Triple-agent *n* = 70 (%)	*p*-value
**Laparoscopic**								
*No*	41 (18.1)	10 (11.0)	31 (22.8)	0.037	29 (20.7)	10 (14.3)	19 (27.1)	0.095
*Yes*	186 (81.9)	81 (89.0)	105 (77.2)		111 (79.3)	60 (85.7)	51 (72.9)	
**Resection extend**								
*Distal*	93 (41.0)	45 (49.5)	48 (35.3)	0.047	59 (42.1)	31 (44.3)	28 (40.0)	0.732
*Total*	134 (59.0)	46 (50.5)	88 (64.7)		81 (57.9)	39 (55.7)	42 (60.0)	
**R0 resection**								
*R0*	202 (89.0)	82 (90.1)	120 (88.2)	0.829	124 (88.6)	62 (88.6)	62 (88.6)	1
*R1 or R2*	25 (11.0)	9 (9.9)	16 (11.8)		16 (11.4)	8 (11.4)	8 (11.4)	
**Complications**^**a**^								
*Overall*	45 (19.82)	8 (8.8)	37 (27.2)	0.001	23 (16.4)	4 (5.7)	19 (27.1)	0.001
*Abdominal infection*	15 (6.6)	1 (1.1)	14 (10.3)	0.014	8 (5.7)	0 (0.0)	8 (11.1)	0.011
*Anastomotic leakage*	12 (5.3)	1 (1.1)	11 (8.1)	0.045	7 (5.0)	0 (0.0)	7 (10.0)	0.020
*Digestive obstruction*	2 (0.9)	1 (1.1)	1 (0.7)	1	1 (0.7)	1 (1.4)	0 (0.0)	1
*Pneumonia*	2 (0.9)	1 (1.1)	1 (0.7)	1	2 (1.4)	1 (1.4)	1 (1.4)	1
*Bleeding*	5 (2.2)	2 (2.2)	3 (2.2)	1	2 (1.4)	1 (1.4)	1 (1.4)	1
*Arrhythmia*	2 (0.9)	1 (1.1)	1 (0.7)	1	1 (0.7)	0 (0.0)	1 (1.4)	1
*Pleural effusion*	7 (3.1)	1 (1.1)	6 (4.4)	0.306	2 (1.4)	1 (1.4)	1 (1.4)	0.612
**Tumor regression grade**								
*Grade 0(complete response)*	32 (14.1)	10 (11.0)	22 (16.2)	0.686	19 (13.6)	8 (11.4)	11 (15.7)	0.642
*Grade 1(major response)*	37 (16.3)	14 (15.4)	23 (16.9)		24 (17.1)	10 (14.3)	14 (20.0)	
*Grade 2(moderate response)*	128 (56.4)	54 (59.3)	74 (54.4)		79 (56.4)	42 (60.0)	37 (52.9)	
*Grade 3(minor response)*	30 (13.2)	13 (14.3)	17 (12.5)		18 (12.9)	10 (14.3)	8 (11.4)	
**Pathological T stage**								
*T0*	35 (15.4)	10 (11.0)	25 (18.4)	0.240	20 (14.3)	8 (11.4)	12 (17.1)	0.783
*T1*	25 (11.0)	11 (12.1)	14 (10.3)		15 (10.7)	8 (11.4)	7 (10.0)	
*T2*	23 (10.1)	13 (14.3)	10 (7.4)		17 (12.1)	10 (14.3)	7 (10.0)	
*T3*	137 (60.4)	56 (61.5)	81 (59.6)		85 (60.7)	43 (61.4)	42 (60.0)	
*T4a*	6 (2.6)	1 (1.1)	5 (3.7)		3 (2.1)	1 (1.4)	2 (2.9)	
*T4b*	1 (0.4)	0 (0.0)	1 (0.7)		–	–	–	
**Pathological N stage**								
*N0*	107 (47.1)	50 (54.9)	57 (41.9)	0.183	73 (52.1)	40 (57.1)	33 (47.1)	0.723
*N1*	48 (21.1)	17 (18.7)	31 (22.8)		25 (17.9)	12 (17.1)	13 (18.6)	
*N2*	38 (16.7)	12 (13.2)	26 (19.1)		21 (15.0)	10 (14.3)	11 (15.7)	
*N3a*	7 (3.1)	4 (4.4)	3 (2.2)		5 (3.6)	2 (2.9)	3 (4.3)	
*N3b*	18 (7.9)	7 (7.7)	11 (8.1)		11 (7.9)	5 (7.1)	6 (8.6)	
**Positive lymph nodes**	1 [0,4]	0 [0,3]	1 [0,5]	0.082	0[0, 4]	0 [0, 2]	1 [0, 5]	0.205
**Harvested lymph nodes**	28 [19,37]	28 [22,37.50]	28 [19,36.25]	0.793	28.5 [19, 38.25]	28 [20.25, 38]	29.5 [19, 39.75]	0.987
**Adjuvant chemotherapy**								
*None*	16 (7.0)	6 (6.6)	10 (7.4)	0.329	10 (7.1)	6 (8.6)	4 (5.7)	0.288
*Single agent regimen*	24 (10.6)	13 (14.3)	11 (8.1)		15 (10.7)	10 (14.3)	5 (7.1)	
*Multiple agents regimen*	187 (82.4)	72 (79.1)	115 (84.6)		115 (82.1)	54 (77.1)	61 (87.1)	

^a^Complications were classified according to Clavien-Dindo system

### Survival analysis

**Fig. 2:**
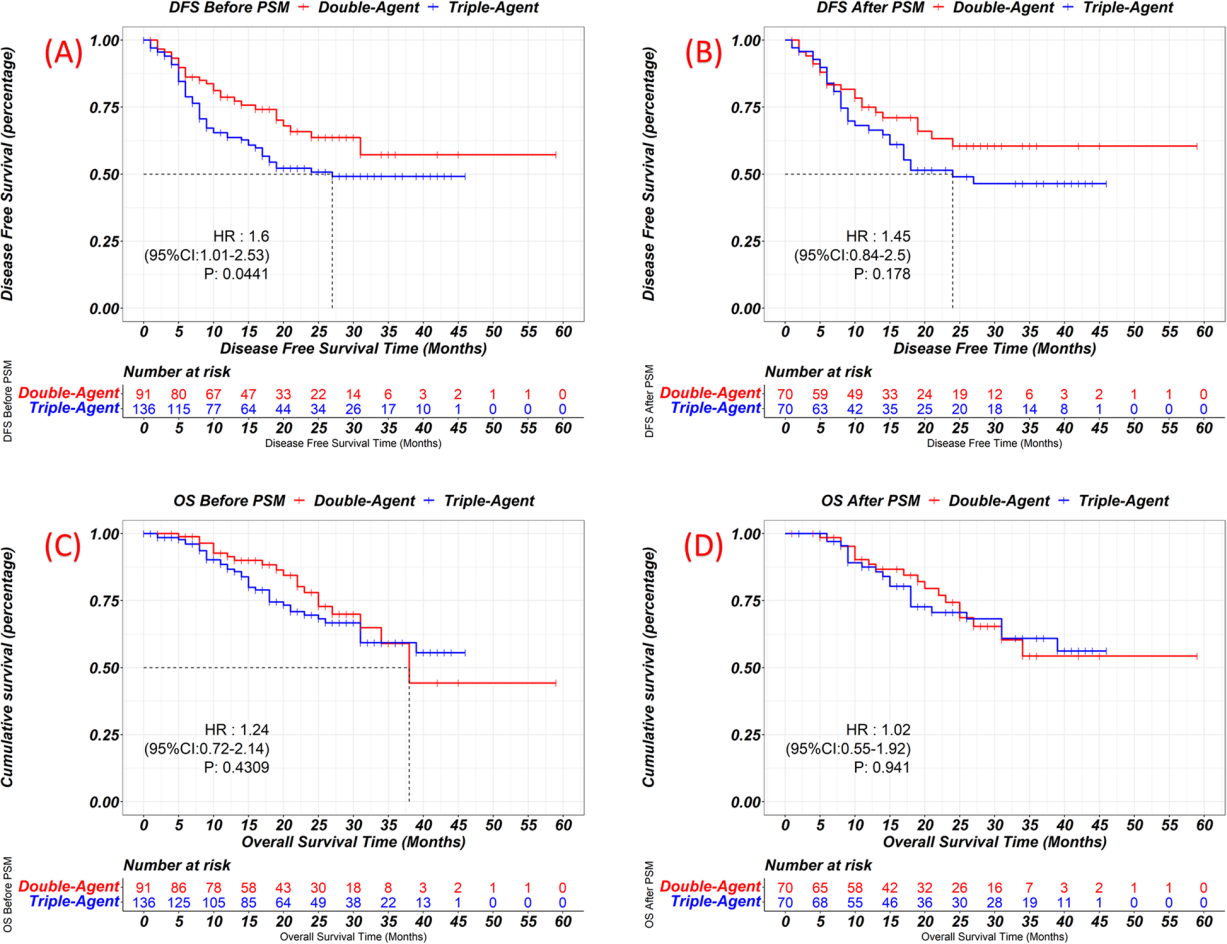
The Kaplan–Meier curves showing the disease free survival of doublet and triplet cohort before (**A**) and after (**B**) PSM, and overall survival before (**C**) and after (**D**) PSM

**Fig. 3:**
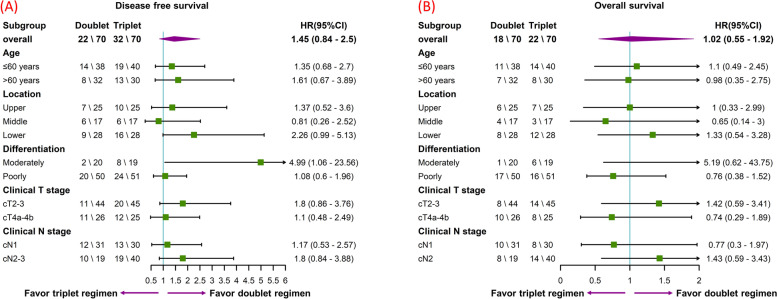
Forest plot showing the subgroup analysis of the disease free survival **(A)** and overall survival **(B)**. Triplet neoadjuvant chemotherapy regimens were correlated with worsen DFS for patients with moderately differentiated adenocarcinoma

The median follow-up time was 31 months. As shown in Fig. 2, before PSM, the disease-free survival (DFS) in the triplet cohort was shorter, but the difference becomes insignificant after PSM (1-year DFS rate, Doublet 77.1% vs. Triplet 68.6%, *p* = 0.178). The overall survival (OS) was similar in both cohorts, before or after PSM (3-years OS rate after PSM, Doublet 54.3% vs. Triplet 60.9%, *p* = 0.941). In subgroup survival analysis (Fig. 3), the triplet cohort also failed to exhibit any superiority in any subgroups. In the subgroup of patients with moderately differentiated adenocarcinoma, triplet NAC was even correlated with shorten DFS. Therefore, these data suggested that the triplet regimen has brought no additional survival benefit compared with the doublet regimen.

## Discussion

The triplet regimens, such as ECF/DCF/FLOT, have been endorsed as NAC regimens for LAGC by many reputable gastric cancer guidelines, such as the NCCN/ESMO. In 2006, the MAGIC trial [18] proved that NAC with epirubicin, cisplatin and fluorouracil (the ECF regimen) improved survival compared with surgery alone, setting ECF as the first recommended NAC regimen. However, many studies have reported that ECF was less tolerable in clinical practice due to the high toxicity profile, limiting its use. Later on, in the FLOT4 [8] study, a triplet regimen consisted of docetaxel, oxaliplatin and fluorouracil (the FLOT regimen) exhibited a more satisfactory efficacy than the ECF regimen, reaching a median survival of 50 months, making it the standard NAC regimen ever since. However, doublet regimens combining oxaliplatin and fluorouracil, such as SOX or CAPOX, are still commonly used as NAC regimens in Asian countries, including China, Japan, and Korea [19–21]. Some preliminary randomized trials showed that these modernized doublet regimens also exhibit satisfactory efficacy [22, 23]. Thus some researchers proposed that these doublet regimens could also be used as first-line NAC regimen, with efficacy that is non-inferior to triplet regimen and lower toxicity profiles. While some insisted that triplet regimens such as FLOT or DCF should be the golden standard because a more intense preoperative chemotherapy could better downstage the tumor, improve R0 resection rate, and eventually prolong survival. Thus, the issue of which should be the most appropriate NAC regimen for LAGC, doublet or triplet, is still left unsettle.

In this study, we retrospectively reviewed 227 cases of LAGC who received double or triplet NAC to investigate the toxicity profile and efficacy. We use PSM to select two cohorts with similar characteristics, making them comparable. The matching factors were sex, age, tumor location, differentiation, diabetes, clinical T/N stage, and clinical stage groupings, all pre-intervention factors related to the treatment outcome and survival. Since the pathological stage may be altered by the NAC, clinical stages are the most important confounding factor for this study [24]. After PSM, the clinical stages were almost identical in the two cohorts. We found that the triplet NAC regimen is not only no better than the doublet regimen in terms of efficacy, it may also potentially bring more post-surgery complications.

Firstly, the most important indicators for efficacy valuation are the R0 resection rate, tumor regression grade (TRG), and survival. TRG is the classification of cancer response to preoperative treatment based on the residual cells remained in the tumor lesions. The best case is pathological complete response (PCR), which means no residual cells remained [25]. TRG is considered a more objective indicator of tumor response than radiological response grade since pseudoprogression is possible when evaluating empty organs on radiological images [26]. Our results showed that the triplet regimens did not bring forth a higher PCR rate, nor a better downstaging, than the doublet regimens. The R0 resection rate was not significantly improved by the triplet regimens either. R0 resection means a more thorough clearance of the tumor and is a major achievement goal of NAC, which is related to prolonged survival. The DFS and OS were not improved in the triplet cohort either. Deteriorated DFS was observed in the subgroup with moderate adenocarcinoma. However, considering the small sample size in each subgroup, this result shall be interpreted with caution.

For the safety evaluation of the regimens, as depicted in Table 2, after PSM, the incidence of anemia was higher in the triplet cohort, most cases required blood transfusion. This might be related to the addition of docetaxel, as a previous study had found that docetaxel induced more severe anemia [27, 28]. Thrombocytopenia was more frequent in the doublet cohort, which could be explained by the increased dosage of oxaliplatin per cycle, as oxaliplatin was more likely to induce thrombocytopenia [29, 30]. The incidence rate of grade 3/4 toxicity that required supportive treatment was similar, though. As for perioperative safety, the Clavien-Dindo grade 2–4 complications incidence rate was higher in the triplet cohort. The most common complication was deep abdominal infection and abscess, caused mainly by anastomotic leakage and fistula. The most likely explanation is that more intense triplet NAC regimen could induce more severe tissue edema and coagulative dysfunction, affecting the healing of the anastomosis, leading to higher risks of post-surgery complications, as proposed by previous studies [31–33]. Another possible explanation is that laparoscopic surgery could be more commonly used in the doublet cohort. Laparoscopic approaches have the benefits over open surgeries through visual magnification, better exposure, and more delicate maneuvers of organs, vessels, and nerves, all of which may contribute to a lower incidence of post-surgery complications [34].

To our knowledge, this is the first study that systematically compares the efficacy and toxicity of doublet and triplet regimens. The usage of PSM has enabled us to balance all the pre-intervention confounding factors, making the result more reliable. However, there were a few limitations to our study. Firstly, the effect of selection bias was not neglectable due to the nature of retrospective studies. Due to the fact that different oncologists might choose different regimens within the scope of the guidelines, it should be noted that the selection bias brought by doctors may be present. Secondly, the relatively small sample size had limited the reliability of the conclusion, especially for the subgroup analysis. Data of patients with the intention of surgery but eventually lost the opportunity due to the progression of the tumor during NAC were not retrievable. All the patients enrolled in the study had already received NAC and sequential surgery. However, these patients only accounted for a very small proportion, so it may not have a major impact on the result. Thirdly, the DCF regimen adopted in our study was not standardized, in which the dosage per cycle was reduced while the intervals between cycles were shortened. But the dosage density in our modified regimen remained the same as the original one, and only a few cases received this modified DCF regimen, which was unlikely to alter the final result. Fourthly, information about pre-intervention laparoscopic staging was lacking, but none of the patients enrolled in our study were found with peritoneal dissemination during the resection surgery, thus it may not alter the final conclusion. Lastly, genetic differences and other perioperative drug use such as low molecular weight heparins may also affect the prognosis, as indicated by other previous studies [35–39]. Thus, more investigations are needed to further confirm the findings.

## Conclusions

Compared with doublet NAC regimens, triplet regimens may not be superior in improving tumor regression grade, R0 resection rate, and survival in patients with LAGC, and may bring a higher risk of post-surgery complications.

## Data Availability

The datasets analysed during the current study are not publicly available due to our institution policy but are available from the corresponding author on reasonable request.

## Abbreviations

NAC: Neoadjuvant chemotherapy
LAGC: Locally advanced gastric cancer
PSM: Propensity score matching
ESMO: The European Society for Medical Oncology
AJCC: American Joint Committee on Cancer staging systems
NCCN: The National Comprehensive Cancer Network guideline
FLOT: Fluorouracil, leucovorin, oxaliplatin, and docetaxel
DCF: Docetaxel, cisplatin, and 5-fluorouracil
ECF: Epirubicin, cisplatin and fluorouracil
SOX: S-1/oxaliplatin
CAPOX: Capecitabine/oxaliplatin
TRG: Tumor regression grade
ECOG: Eastern Cooperative Oncology Group
FOLFOX: Fluorouracil and oxaliplatin
CT: Computerized tomography
DFS: Disease-free survival
OS: Overall survival
